# Dissemination of clonal complex 2 *Acinetobacter baumannii* strains co-producing carbapenemases and 16S rRNA methylase ArmA in Vietnam

**DOI:** 10.1186/s12879-015-1171-x

**Published:** 2015-10-15

**Authors:** Tatsuya Tada, Tohru Miyoshi-Akiyama, Kayo Shimada, Tran Thi Thanh Nga, Le Thi Anh Thu, Nguyen Truong Son, Norio Ohmagari, Teruo Kirikae

**Affiliations:** Department of Infectious Diseases, Research Institute, National Center for Global Health and Medicine, Shinjuku, Tokyo Japan; Pathogenic Microbe Laboratory, Research Institute, National Center for Global Health and Medicine, Shinjuku, Tokyo Japan; Cho Ray Hospital, Ho Chi Minh, Vietnam; Disease Control and Prevention Center, Division of Infectious Diseases, National Center for Global Health and Medicine, 1-21-1 Toyama, Shinjuku, Tokyo 162-8655 Japan

**Keywords:** Multidrug-resistance, Acinetobacter baumannii, 16S rRNA methylase ArmA, Metallo-β-lactamase NDM-1, Intensive care unit

## Abstract

**Background:**

*Acinetobacter baumannii* strains co-producing carbapenemase and 16S rRNA methylase are highly resistant to carbapenems and aminoglycosides.

**Methods:**

Ninety-three isolates of multidrug-resistant *A. baumannii* were obtained from an intensive care unit in a hospital in Vietnam. Antimicrobial susceptibility tests and whole genome sequencing were performed. Multilocus sequence typing and the presence of drug resistant genes were determined and a maximum-likelihood phylogenetic tree was constructed by SNP alignment of whole genome sequencing data.

**Results:**

The majority of isolates belonged to clonal complex 2 (ST2, ST570 and ST571), and carried carbapenemase encoding genes *bla*_OXA-23_ and *bla*_OXA-66_. Two isolates encoded carbapenemase genes *bla*_NDM-1_ and *bla*_OXA-58_ and the 16S rRNA methylase encoding gene *armA* and did not belong to clonal complex 2 (ST16).

**Conclusion:**

*A. baumannii* isolates producing 16S rRNA methylase ArmA and belonging to clonal complex 2 are widespread, and isolates co-producing NDM-1 and ArmA are emerging, in medical settings in Vietnam.

**Electronic supplementary material:**

The online version of this article (doi:10.1186/s12879-015-1171-x) contains supplementary material, which is available to authorized users.

## Background

Metallo-β-lactamases (MBLs) confer reduced susceptibility to carbapenems, cephalosporins, and all penicillins except monobactams [1]. Acquired MBLs are produced by several Gram-negative bacterial strains, including *Acinetobacter* spp., *Pseudomonas aeruginosa*, and several Enterobacteriaceae [1]. MBLs are categorized by their amino acid sequences into various types [2–4], including AIM [5], DIM [6], FIM [7], GIM [8], IMPs [9], KHM [10], NDMs [11], SMB [12], SIM [13], SPM [14], TMBs [15] and VIMs [16]. The most prevalent MBLs are IMP-, VIM-, and NDM-type enzymes [1, 2, 17]. NDM-1 was initially isolated from *Klebsiella pneumoniae* and *Escherichia coli* in 2008 in Sweden [11]. Between 2009 and 2012, 950 isolates of NDM-1-producing bacteria, including 36 *A. baumannii* isolates, were reported worldwide [18]. Subsequently, at least 13 NDM variants (www.lahey.org/studies) have been reported in several countries [4, 19–30].

Aminoglycosides are effective antibiotics for the treatment of infectious diseases caused by Gram-negative bacteria. These agents block bacterial protein synthesis by binding to the 30S ribosomal subunit [31]. Methylation of 16S rRNA by 16S rRNA methylases, however, makes Gram-negative bacteria highly resistant to all clinically important aminoglycosides [32]. In 2003, clinical isolates of highly aminoglycoside-resistant Gram-negative bacteria producing 16S rRNA methylases were identified in France [33] and Japan [34]. Since then, 16S rRNA methylase-producing Gram-negative bacteria have been isolated in other parts of the world, including Asian countries, such as Afghanistan, Bangladesh, China, Hong Kong, India, Japan, Korea, Oman, and Pakistan [35].

## Methods

### Bacterial samples and drug susceptibility tests

From 2011 to 2013, 93 clinical isolates of *A. baumannii* were obtained from respiratory tract samples taken from patients hospitalized in an intensive care unit (ICU) in Cho Ray Hospital in Ho Chi Minh City, Vietnam.

MICs of amikacin, arbekacin, ciprofloxacin, colistin, imipenem, meropenem, and tigecycline were determined using the microdilution method, as described [36].

### Whole genome sequences

Genomic DNA from the 93 multidrug-resistant isolates were extracted using DNeasy Blood & Tissue kits (QIAGEN, Tokyo, Japan) and sequenced by MiSeq (Illumina, San Diego, CA). MiSeq data, including total length, number of contig, *N*50, average contig length and % GC content, were shown in Additional file 1: Table S1. To identify SNPs among these genomes, all reads of each isolate were aligned against the *A. baumannii* TYTH-1 sequence (Accession no. CP003856) using CLC genomics workbench, version 5.5 (CLC bio, Tokyo, Japan). SNP concatenated sequences were aligned by MAFFT (http://mafft.cbrc.jp/alignment/server/). A maximum-likelihood phylogenetic tree was constructed from the SNP alignment with PhyML 3.0 [37]. The probability of node branching was evaluated with 100 bootstrappings. Raw reads of all isolates were assembled into more than 500 bp contigs by CLC genomics workbench. Contigs around drug-resistant genes were annotated using the BLAST database (http://blast.ncbi.nlm.nih.gov/Blast.cgi?CMD=Web&PAGE_TYPE=BlastHome). Multilocus sequence typing (MLST) based on contig data was deduced using CLC genomics workbench, and matched against the Institut Pasteur MLST (http://pubmlst.org/abaumannii/) databases. The result of STs according to PubMLST (http://pubmlst.org/abaumannii/) scheme was shown in Additional file 2: Table S2. Annotations using the RAST server (http://rast.nmpdr.org/) were performed to compare numbers of prophages and resistance factors. All raw read data of the 93 isolates have been deposited at GenBank as accession numbers DRX032164 to DRX032256.

### Ethical approval

The study protocol was carefully reviewed and approved by the ethics committee of Cho Ray Hospital (approval number: 1644/QD-BVCR), the ethics committee of the National Center for Global Health and Medicine (No. 1268), and the Biosafety Committee of the National Center for Global Health and Medicine (approval number: 27-M-52), respectively. Individual informed consent was waived by the ethics committee listed above because this study used currently existing sample collected during the course of routine medical care and did not pose any additional risks to the patients.

## Results

### Drug susceptibility tests

The majority of the *A. baumannii* isolates tested were highly resistant to carbapenems, aminoglycosides, and ciprofloxacin, but sensitive to colistin and tigecycline (Table 1). MICs were 0.5 – > 512 μg/mL (MIC_50_ > 512 μg/mL and MIC_90_ > 512 μg/mL) to amikacin, 32– > 512 μg/mL (MIC_50_ > 512 μg/mL and MIC_90_ > 512 μg/mL) to ciprofloxacin, 0.125–16 μg/mL (MIC_50_ = 0.5 μg/mL and MIC_90_ = 1 μg/mL) to colistin, 8–128 μg/mL (MIC_50_ = 32 μg/mL and MIC_90_ = 64 μg/mL) to imipenem, 4 to 128 μg/mL (MIC_50_ = 32 μg/mL and MIC_90_ = 64 μg/mL) to meropenem, and < 0.125–16 μg/mL (MIC_50_ = 1 μg/mL and MIC_90_ = 8 μg/mL) to tigecycline. The isolate NCGM321 was particularly resistant to carbapenems and aminoglycosides, with MICs of > 512 μg/mL to amikacin, > 512 μg/mL to arbekaein, 512 μg/mL to ciprofloxacin, 0.25 μg/mL to colistin, 128 μg/mL to imipenem, 64 μg/mL to meropenem, and 2 μg/mL to tigecycline.

**Table 1:** MIC_50_ and MIC_90_ values and antimicrobial resistance of the 93 *A. baumannii* isolates

Antimicrobial agents	Breakpoint for resistance (mg/L)	% Resistant	Range (mg/L)	MIC_50_ (mg/L)	MIC_90_ (mg/L)
Imipenem	≥8	100	8–128	32	64
Meropenem	≥8	99	4–128	32	64
Amikacin	≥64	87	0.5 – > 512	>512	>512
Ciprofloxacin	≥4	100	32 – > 512	>512	>512
Colistin	≥4	5	0.125–16	0.5	1
Tigecycline	-	-	≤0.125–16	1	8

### Molecular epidemiology and drug resistant genes

**Fig. 1:**
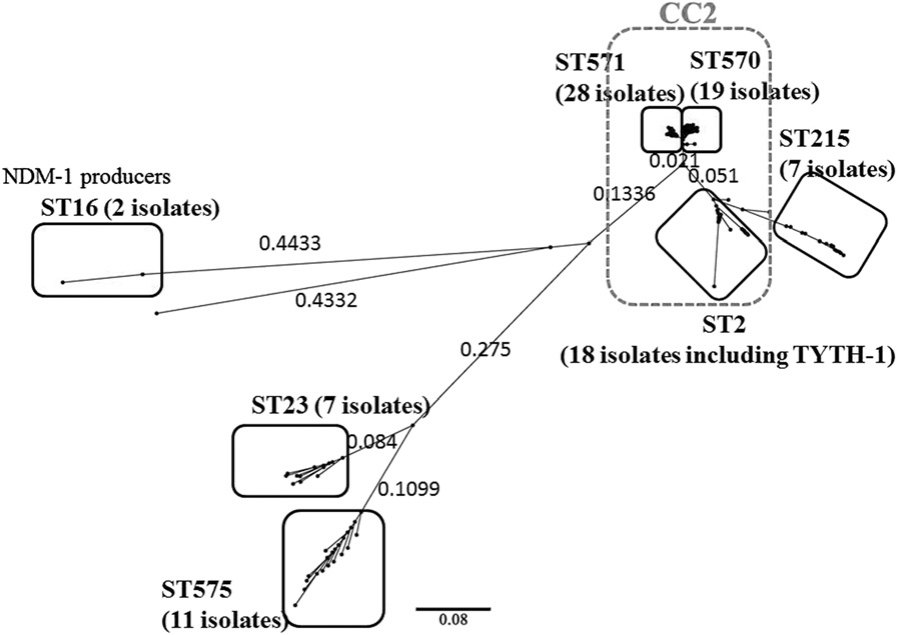
Molecular phylogeny of the 93 *Acinetobacter baumannii* strains from patient isolates. Phylogenic analysis based on SNP concatenation revealed seven clades, ST2, ST16, ST23, ST214, ST570, ST571, and ST575. Isolates in Clades ST2, ST570, and ST571 belonged to clonal complex 2 (CC2)

Phylogenic analysis based on SNP concatenation showed that the 93 isolates belonged to seven clades, ST2 (28 isolates), ST16 (two isolates), ST23 (seven isolates), ST215 (seven isolates), ST570 (19 isolates), ST571 (28 isolates), and ST575 (11 isolates) (Fig. 1). The isolates in Clades ST2, ST570, and ST571 belonged to worldwide clonal lineage II (CC2, European Clone II) [38]. All isolates tested contained intrinsic *bla*_ADC_. No novel *bla*_ADC_ gene was detected. None of the intrinsic *bla*_ADC_ genes contained *ISAba1*, which is responsible for the overexpression of these genes [39]. The intrinsic *bla*_ADC_ genes encoded clade-specific *bla*_OXA-51-like_ variants, with the 71 isolates belonging to Clades ST2, ST215, ST570, and ST571 having *bla*_OXA-66_, the 11 isolates belonging to ST575 having *bla*_OXA-144_, the seven isolates belonging to ST23 having *bla*_OXA-68_, and the two isolates belonging to ST16 having *bla*_OXA-51_ (Table 2). The 2 isolates belonging to ST16 also contained the *bla*_NDM-1_, *bla*_OXA-58_, and *bla*_VEB-1_ genes. Of the all 93 isolates tested, 71 had *bla*_TEM-1_, 56 had *bla*_OXA-23_, and three had *bla*_VEBs_.

**Table 2:** MLST and drug resistance genes in *A. baumannii* isolates

MLST	No. of isolates^*a*^	Carbapenemase and ESBL encoding genes	Aminoglycoside-resistance genes
ST2 (CC2)	17	*bla*_OXA-66_, *bla*_OXA-23_ (9/17), *bla*_PER-1_ (1/17), *bla*_TEM-1_ (16/17)	*armA*, *aac(6’)-Ib-cr* (13/17), *aac(3)-Ia*(1/17), *aadA1* (14/17), *aph(3’)-Ia* (14/17)
ST16	2	*bla*_NDM-1_, *bla*_OXA-51_, *bla*_OXA-58_, *bla*_VEB-1_, *bla*_TEM-1_ (1/2)	*armA* (1/2), *aac(3)-Iid*, *aadA1*, *aadB*, *aph(3’)-*Via
ST23	7	*bla*_OXA-23_ (2/7), *bla*_OXA-68,_ *bla*_PER-1_, *bla*_TEM-1_ (1/23)	*armA* (3/7), *aac(6’)-Ib-cr* (4/7), *aadA1* (3/7), *aadB* (1/7), *aph(3’)-Ia* (1/7), *aph(3’)-*Via (5/7), *aph(3’)-Vib* (1/7), *aphA6* (1/7)
ST215	7	*bla*_OXA-23_ (4/7), *bla*_OXA-66_, *bla*_TEM-1_	*armA* (6/7), *aac(6’)-Ib* (1/7), *aac(6’)-Ib-cr* (5/7), *aac(3)-Ia* (4/7), *aadA1*, *aph(3’)-Ia* (5/7)
ST570 (CC2)	19	*bla*_OXA-23_ (9/19), *bla*_OXA-66,_ *bla*_TEM-1_ (18/19)	*armA*, *aac(6’)-Ib* (7/19), *aac(6’)-Ib-cr* (11/19), *aac(3)-Ia*, *aadA1*, *aph(3’)-Ia* (7/19)
ST571 (CC2)	28	*bla*_OXA-23_ (22/28), *bla*_OXA-66,_ *bla*_PER-1_ (5/28), *bla*_TEM-1_	*armA*, *aac(3)-Iid* (4/28), *aadA1, aadB (2/28)*, *aph(3’)-*Via (1/28), *aph(3’)-Vib* (4/28)
ST575	11	*bla*_OXA-23_ (9/11), *bla*_OXA-144,_ *bla*_PER-1_, *bla*_TEM-1_	*aadB* (5/11), *aph(3’)-*Via (3/11), *aph(3’)-Vib* (4/11), *aphA6* (4/11)
ST577	1	*bla*_OXA-23_, *bla*_OXA-66,_ *bla*_TEM-1_	*armA*, *aac(6’)-Ib*, *aac(3)-Ia*, *aadA1*
ST578	1	*bla*_OXA-51,_ *bla*_OXA-58_, *bla*_VEB-7_	*aac(3)-Ib*, *aadA1*, *aph(3’)-*Via

^*a*^Total number of isolates belonging to the same sequence type

Among the 93 isolates, 77 had *armA*, 77 had *aadA1*, 34 had *aac(6’)-Ib-cr*, 28 had *aph(3’)-Ia*, 18 had *aac(3)-Ia*, 12 had *aph(3’)-*Via, 10 had *aph(3’)-Vib*, seven had *aac(3)-Iid*, five had *aphA6*, and one had *aac(3)-Ib*. No plasmid was detected in any of the 93 isolates, indicating that all drug resistance genes were located on chromosomes.

### Genomic environments surrounding *armA*, *bla*_OXA-23_*bla*_NDM-1_, *bla*_OXA-51-like_, and *bla*_OXA-58_

**Fig. 2:**
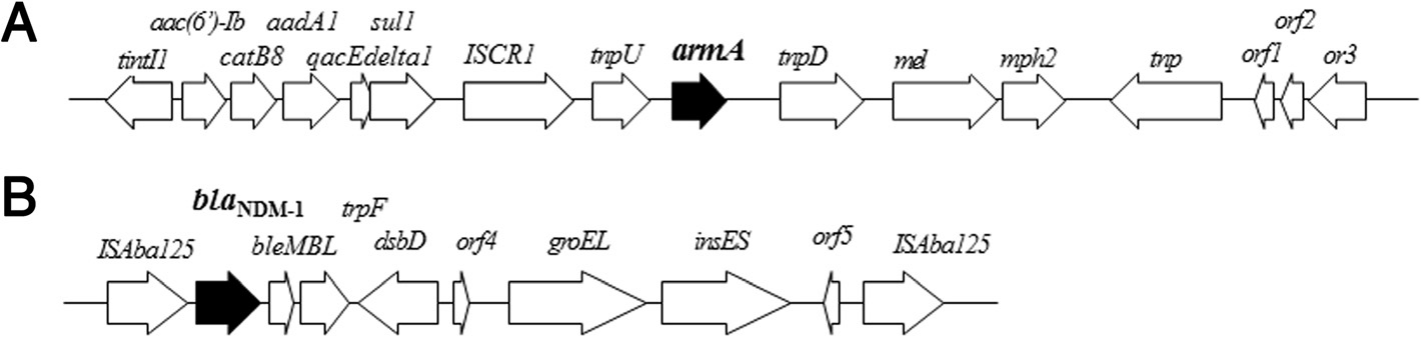
Genetic environments of *armA* and *bla*_NDM-1_ in *A. baumannii* NCGM346 (**a**) and NCGM321 (**b**). *orf1*, gene encoding a hypothetical protein; *orf2*, gene encoding a DNA-binding protein; *orf3*, gene encoding a DNA replication protein; *orf4* and *orf5*, genes encoding hypothetical proteins

The genetic environment surrounding *armA* in NCGM346 belonging to Clade ST571 (Accession no. LC030435) is shown in Fig. 2a. This genetic environment, from nt 1 to nt 17,473, was more than 99.99 % homologous to the analogous region of *A. baumannii* strains MDR-TJ isolated in China [40] and NCGM253 isolated in Japan [41]. The sequence surrounding *armA* from nt 5838 to nt 9879 was identical to the transposon Tn*1548* (Accession no. EU014811) detected in an *A. baumannii* isolate from North America [42] and included the IS*CR1* insertion sequence. Putative transposase genes were located both upstream (*tnpU*) and downstream (*tnpD*) of *armA* (Fig. 2a). Four additional isolates, NCGM165, NCGM169, NCGM175, and NCGM194, belonging to Clades ST570, ST215, ST23, and ST2, respectively, had the same genetic organization surrounding *armA* as the NCGM346 isolate. None of these five isolates contained plasmids, indicating that *armA* is chromosomally encoded in each.

The genetic environment surrounding *bla*_NDM-1_ in NCGM321 belonging to Clade ST16 (Accession no. LC032101) is shown in Fig. 2b. The *bla*_NDM-1_ gene was located between two copies of *ISAba125* and was carried by the Tn125 composite transposon. The genetic environment surrounding *bla*_NDM-1_ was 100 % homologous to those of *A. baumannii* strain IOMTU433 isolated in Nepal (accession no. AP014649), *A. baumannii* ZW85-1 plasmid pAbNDM-1 isolated in China (accession no. JN377410), *Acinetobacter lwoffii* WJ10621 plasmid pNDM-BJ01 isolated in China (accession no. JQ001701), and *A. baumannii* 161/07 isolated in Germany (accession no. HQ857107). The genetic environment surrounding *bla*_NDM-1_ in NCGM328, the second isolate belonging to Clade ST16, was identical to the genetic environment surrounding *bla*_NDM-1_ in NCGM321.

The genetic environment surrounding *bla*_OXA-23_ in NCGM346 belonging to Clade ST571 was *ISAba1-bla*_OXA-23_-*yeeA* (*yeeA*: ATPase encoding gene) and was more than 99 % identical with chromosome sequences of *A. baumannii* strains IOMTU433 (accession no. AP014649) and NCGM237 [41]. The genetic organization surrounding *bla*_OXA-23_ in four additional isolates, NCGM165, NCGM169, NCGM175, and NCGM194, belonging to Clades ST570, ST215, ST23, and ST2, respectively, was identical to that surrounding *bla*_OXA-23_ in NCGM346.

The genetic environment surrounding *bla*_OXA-51-like_ in NCGM346 belonging to Clade ST571 was *fxsA*-*bla*_OXA-66_-*orf6*-*orf7*-*ruvC*-*orf8*-*gueG*-*bioB*, where *orf6* encodes the enzyme phosphinothricin N-acetyltransferase, *orf7* encodes an XRE family transcriptional regulator, and *orf8* encodes a hypothetical protein. The same genetic organization surrounding *bla*_OXA-51-like_ was observed in four additional isolates, NCGM165, NCGM169, NCGM175, and NCGM194, belonging to Clades ST570, ST215, ST23, and ST2, respectively.

The genetic environment surrounding *bla*_OXA-58_ in NCGM328 belonging to Clade ST16 was *ISAba3*-*bla*_OXA-58_-*orf9*-*orf10*-*ISAba3*, where *orf9* encodes a transposon-related protein and *orf10* encodes a hypothetical protein. The structure was the same as a part of *Acinetobacter* spp. M131 plasmid pM131-2 (accession no. JX101647).

### Structures of the genomic resistance islands of CC2 isolates

The resistance island (RI) of the isolate NCGM196 belonging to Clade ST2 contained two Tn6021-like copies and one Tn5393-like copy. The resistance genes in the RI included *sul1*, which encodes sulfonamide resistance protein, and *tetB* and *tetR*, which regulate tetracycline resistance, as well as the streptomycin resistance genes *strA* and *strB*. The RI structure of the other ST2 isolate (NCGM194) was identical to that of *A. baumannii* MDT-TJ [40] and TYTH-1 [43]. RIs of the isolates belonging to Clades ST570 (NCGM165) and ST571 (NCGM346) were identical to those of AbaR4 [44], a compound transposon containing a Tn6022 backbone.

### Prophages and resistance factors

The *A. baumannii* isolates had several transposable elements, phages/prophages and resistance factors. The isolates belonging to international clone 2, including NCGM165 (ST570), NCGM194 (ST2) and NCGM346 (ST571), had fewer phages/prophages than the isolates belonging to other clones, including NCGM169 (ST215), NCGM175 (ST23) and NCGM328 (ST16). The isolates NCGM165, NCGM194, NCGM346, NCGM169, NCGM175, and NCGM328 contained 10, 18, 10, 8, 7, and 6 transposable elements, respectively; 54, 60 80, 57, 57 and 57 resistance factors, respectively; and 11, 8, 8, 27, 49 and 32 phages/prophages, respectively.

## Discussion

To our knowledge, this is the first report of *A. baumannii* isolates co-producing NDM-1 and ArmA emerging in a medical setting in Vietnam. Enterobacteriaceae producing only NDM-1 had been reported in Vietnam [18, 45, 46], including NDM-1-producing *K. pneumoniae* isolated from environmental samples [45] and NDM-1-producing Enterobacteriaceae isolated from samples in a Vietnamese surgical hospital [47]. There have been no reports of *A. baumannii* co-producing NDM-1 and ArmA and belonging to international clone 2, although NDM-1 producers belonging to international clone2 were reported in East Africa in 2013 [48]. It is important to continue the surveillance of NDM-1-producing pathogens, including *A. baumannii*, in medical settings in Vietnam.

The high prevalence of Gram-negative bacteria producing ArmA in Vietnam may result from the inadequate use of aminoglycosides in that country. An analysis of patients hospitalized in Vietnam showed that 67.4 % received antibiotics, with 18.9 % receiving aminoglycosides, although 30.8 % of the prescribed antibiotics were considered inappropriate [49]. This latter rate was higher than the rates of inappropriately prescribed antibiotics in Malaysia (4.0 %) [50], Turkey (14.0 %) [51], Hong Kong (20.0 %) [52] and European countries (17.8–32.0 %) [53, 54].

A similar genetic environment surrounding *bla*_NDM-1_ has been reported in *A. baumannii* stains isolated in China [55], Colombia (accession no. CP010399), France [22], Germany [56] and the United States (accession no. CP010370); in *A. lwoffii* isolated in China [57]; in *E. coli* isolated in Colombia (accession no. CP010373); in *K. pneumoniae* isolated in Colombia (CP010391) and the United States [58]; and in *Providencia rettgeri* isolated in Canada [59]. A similar environment surrounding *armA* was reported in *A. baumannii* strains isolated in China [40], Japan [41], and Nepal (accession no. AP014649). The genetic organization of *bla*_NDM-1_ has spread worldwide, whereas that of *armA* has spread in Asian countries.

*A. baumannii* isolates belonging to international clone 2 must have been disseminated throughout medical settings in Vietnam, since 69.9 % of all isolates tested belonged to this clone (ST2, ST570, and ST571). Epidemiological studies of *A. baumannii* isolates obtained from a hospital in Hanoi are currently ongoing to clarify whether *A. baumannii* isolates belonging to international clone 2 are disseminating throughout Vietnam. The isolates belonging to Clades ST16, ST23, and ST215 were not identified as belonging to any previously described international clones [38]. To date, one *A. baumannii* isolate belonging to Clade ST16 was isolated in 2001 in the Netherlands, 3 isolates belonging to ST23 were isolated in the Netherlands (in 1964) and Sweden (in 2006 and 2007), and 6 isolates belonging to Clade ST215 were isolated in 2008 in China. Clones ST570, ST571, and ST575 were novel STs. Of the isolates belonging to CC2, those in Clades ST570 and ST571 may have evolved in a unique manner in Vietnam because the structures of resistant islands in ST570 and ST571 isolates were different from those in ST2 isolates.

## Conclusions

This study showed that 16S rRNA methylase ArmA-producing *A. baumannii* isolates belonging to clonal complex 2 have spread, and that NDM-1-and ArmA-co-producers not belonging to clonal complex 2 are emerging, in medical settings in Vietnam.

## Additional files

Additional file 1: Table S1. Assembly summary report of 93 *A. baumannii* isolates using CLC Genomics Workbench version 5.5. (XLSX 15 kb) Additional file 2: Table S2. MLST analysis on PubMLST scheme in 93 *A. baumannii* isolates. (XLSX 14 kb)

## Abbreviations

MBLs: Metallo-β-lactamases
MLST: Multilocus sequence typing
IP-MLST: Institut Pasteur MLST
